# Mini-Incision versus Standard Incision Total Hip Arthroplasty Regarding Surgical Outcomes: A Systematic Review and Meta-Analysis of Randomized Controlled Trials

**DOI:** 10.1371/journal.pone.0080021

**Published:** 2013-11-12

**Authors:** Chang-Peng Xu, Xue Li, Jin-Qi Song, Zhuang Cui, Bin Yu

**Affiliations:** 1 Department of Orthopaedics and Traumatology, Nanfang Hospital, Southern Medical University, Guangzhou, Guangdong, People’s Republic of China; 2 Key Laboratory of Bone and Cartilage Regenerative Medicine, Nanfang Hospital, Southern Medical University, Guangzhou, Guangdong, People’s Republic of China; University of Louisville, United States of America

## Abstract

**Purpose:**

It remains controversial whether mini-incision (MI) benefits patients in total hip arthroplasty (THA). We performed a meta-analysis of randomized controlled trials (RCTs) to assess the effects of MI on surgical and functional outcomes in THA patients.

**Methods:**

A systematic electronic literature search (up to May 2013) was conducted to identify RCTs comparing MI with standard incision (SI) THA. The primary outcome measures were surgical and functional outcomes. According to the surgical approach taken, MI THA patients were divided into four subgroups for sub-group meta-analysis. Standardized mean differences (SMDs) or risk differences (RDs) with accompanying 95% confidence intervals (CIs) were calculated and pooled using a fixed-effect or random-effect model according to the heterogeneity.

**Results:**

A total of 14 RCTs involving THA 1,174 patients met the inclusion criteria. The trials were medium risk of bias. The overall meta-analysis showed MI THA reduced total blood loss (95% CI, -201.83 to -21.18; p=.02) and length of hospital stay ( 95% CI, -0.67 to -0.08; p=.01) with significant heterogeneity. However, subgroup meta-analysis revealed posterior MI THA had perioperative advantages of reduced surgical duration ( 95% CI, -8.45 to -2.67; P<.001), less blood loss ( 95% CI, -107.20 to -1.73; P=.04) and shorter hospital stay ( 95% CI, -0.74 to -0.06; p=.002) with low heterogeneity. There were no significant differences between MI and SI THA groups in term of pain medication dose, functional outcome (HHS), radiological outcome or complications (P>.05, respectively).

**Conclusions:**

Although no definite overall conclusion can be arrived at on whether MI THA is superior to SI THA, posterior MI THA clearly result in a significant decrease in surgical duration, blood loss and hospital stay. It seems to be a safe minimally invasive surgical procedure without increasing the risk of component malposition or complications.

## Introduction

The consequences of introducing mini-incision (MI) into total hip arthroplasty (THA) are still a debating topic in all orthopedic forums [1]. Despite a large amount of existing papers, there are hardly any well designed trials capable of giving a conclusion, based on high-level evidence, on whether MI THA is superior to SI THA. MI is here defined as the use of a 10 cm or even smaller incision to complete the total hip joint replacement [2,3]. 

Advantages of MI THA were reported as less soft tissue trauma (smaller skin incision and less muscle damage), reduced blood loss and fewer blood transfusion requirements. Postoperative benefits that have been demonstrated in some studies include less pain, shorter hospital stay, quicker return to function and a better cosmetic appearance. However, many studies believe that MI THA introduces additional risks due to its limited visibility of anatomical landmarks and vital structures [4]. Some have shown that MI THA is more prone to complications, mainly due to component malpositioning with an increased risk of dislocation, in addition to an increased risk of neurovascular complications and excessive skin trauma [5]. Another drawback seems to be the learning curve which tends to be longer for surgeons with little experience of hip prosthetic surgery. 

For quite a long time, comparison of MI versus SI THA has been addressed by a number of randomized control studies and several meta-analyses [3,6–11], but their findings are still inconsistent. Moskal et al. [7] in their meta-analysis concluded that short-term recovery favored MI over SI THA. Li et al. [6] indicated that MI THA was not superior to SI THA in early postoperative recovery, hip function, and complication rate. In addition, Smith et al. [9] showed that MI THA was associated with a significantly increased risk of transient palsy of the lateral femoral cutaneous nerve but with no significantly better outcome. 

These analyses, however, took all the MI THA as a whole in the comparison with SI THA, regardless of their difference in surgical approach [12]. Consequently, their results usually had a high heterogeneity, compromising the power of their findings. We believe it is probable that surgical approaches have a definite effect on the surgical and functional outcomes of THA because different approaches affect different anatomical structures, leading to different complications. Clearly, a 10 cm posterior approach would have very different risks and complications than an 8 cm direct anterior, or a two-incision approach. Therefore, it is rational to explore the effect of surgical approaches on the outcomes of THA. One recent meta-analysis noticed this problem and grouped their patients pooled into four divisions according to the four different approaches used in THA. However, they addressed only radiological outcomes and complications in comparison of MI and SI THA [13]. Importantly, evidence pertaining to surgical and functional outcomes and long-term implant durability is lacking [14]. In addition, the published meta-analyses comparing MI THA versus SI THA included non-randomised studies and the randomised clinical trails (RCTs) they included were quite limited in number. 

In order to clarify whether MI THA is superior to SI THA in general or in a specific surgical approach used in THA in terms of surgical and functional outcomes on the basis of new evidence, we conducted the present meta-analysis of all the RCTs available up-to-date in English. In the present study sub-group analyses were performed on the basis of THA surgical approaches. 

## Methods

### Search Strategy

This study followed the Preferred Reporting Items for Systematic Reviews and Meta-Analyses (PRISMA) statement (Checklist S1). PubMed, Cochrane Library, EMBASE, BIOSIS and Ovid databases (up to May 2013) were searched to identify RCTs comparing MI THA and SI THA. The structured search strategies used the following search terms: (‘minimally invasive’ or ‘less invasive’ or ‘minimal incision’ or ‘mini-incision’ or ‘MIS’) and ‘hip’ and (‘replacement’ or ‘arthroplasty’ or ‘THR’ or ‘THA’). The search was limited to human subjects and RCTs published in English language. In addition, the reference lists of identified studies were manually checked to identify other potentially eligible trials. This process was performed iteratively until no additional articles could be identified.

### Inclusion and Exclusion Criteria

To make sure the studies are clinically homogeneous, trials were considered acceptable for inclusion in the present meta-analysis if they met the following criteria in PICOS order: (i) population, patients receiving THA in MI and SI groups that were demographically similar and had no statistically significant differences with respect to the variables of age, gender, body mass index (<35.0); (ii) intervention, MI THA via the posterior, posterolateral, lateral or anterolateral approaches; (iii) comparison intervention, THA with a standard or conventional surgery; (iv) outcome measures, 1 or more of the following outcomes reported: surgical outcomes, functional outcomes, radiological outcomes, and complications; and (v) study design, RCT. 

Trials were excluded if they (1) were abstracts, letters, or meeting proceedings; (2) used repeated data or did not report outcomes of interest; and (3) enrolled MI THA that employed computer navigation system or multiple incisions rather than a single surgical exposure.

### Data Extraction and Outcome Measures

Two authors independently extracted the following data: first author, year of publication, number of patients, patient characteristics, study design/risk of bias, MI group (surgical approach and incision length), SI group (surgical approach and incision length), and outcomes. Extracted data were entered into a standardized Excel file. Any disagreements were resolved by discussion and consensus. 

The surgical outcome measurements included surgical duration, blood loss, pain and length of hospital stay. The functional outcome measurement was Harris Hip Score (HHS). The radiological outcomes included outliers of cup abduction, cup anteversion and stem position which varied across studies, and leg-length discrepancy. Complications included incidence of dislocation, nerve injury, infection, deep vein thrombosis (DVT), proximal femoral fracture, component loosening, revision and heterotopic ossification. 

### Risk-of-Bias Quality Assessment and Quality Scoring

Risk-of-bias assessment was performed in accordance with the guidelines outlined in the Cochrane Handbook for Systematic Reviews of Interventions (version 5.1.0) [15]. The Cochrane Collaboration’s tool for assessing risk of bias is illustrated in Table S1. The studies included were assessed and assigned a value of high, low, or unclear as follows: high (risk of bias): one or more of the criteria met; low (risk of bias): all the criteria met; unclear (moderate risk of bias): one or more of the criteria partly met. 

The quality scoring for each trial was given using the modified Jadad scale [16]. It is an eight-item scale designed to assess randomization, blinding, withdrawals and dropouts, inclusion and exclusion criteria, adverse effects and statistical analysis which is illustrated in Table S2. The score for each article can range from 0 (lowest quality) to 8 (highest quality). Scores of 4 to 8 denotes good to excellent quality and 0 to 3 poor to low quality. Two reviewers independently assessed each RCT and any disagreements were resolved by discussion and consensus.

### Statistical Analysis

Standardized mean differences (SMDs) with 95% confidence intervals (CIs) for continuous outcomes and differences were expressed as risk differences (RDs) with 95% CIs for dichotomous outcomes. For the data which were published as median, rang and the size of the trial, MD and SD were calculated by the method of Hozo [17]. Heterogeneity across studies was tested using the I^2^ statistic, which is a quantitative measure of inconsistency across studies. Studies with an I^2^ statistic of 25%–50% are considered to have low heterogeneity, those with an I^2^ statistic of 50%–75% have moderate heterogeneity, and those with an I^2^ statistic of >75% have a high degree of heterogeneity [18]. An I^2^ value greater than 50% indicates significant heterogeneity [19]. Generally, a fixed-effects model was used to combine data, and a random-effects model was used in the case of significant heterogeneity (I^2^ > 50%). Because patient characteristics, study designs, and other confounding factors were not consistent between studies, we further conducted sensitivity analyses to explore possible explanations for heterogeneity and to examine the influence of various exclusion criterions on the overall pooled estimate. Our subgroup analyses were conducted according to different surgical approaches used in MI and SI THA. It is noticeable that the subgrouping criteria were fundamentally based on the MI approaches except that one trail used two inconsistent but similar approaches between SI and MI subgroups (Table 1). 

**Table 1:** Main characteristics of RCTs included in the meta-analysis.

Study	Sample Size, No.		THA Type, No.		Mean Age, y		Gender (M/F), No.		Preoperative Diagnosis		BMI, kg/m^2^		Interventions (approach/length, cm)		Jadad score	FU
	Pts	Hips	MI	SI	MI	SI	MI	SI	MI	SI	MI	SI	MI	SI		
Chimento 2005	60	60	28	32	67.2±8.6	65.6±10.5	16/12	13/19	100% OA	100% OA	25.2±3.1	24.8±2.5	PL/8	PL/15	6	2 y
Dorr 2007	60	60	30	30	70.3±9.7	63.9±13.6	17/13	14/16	90% OA	83% OA	27.6±4.5	30.2±6.5	P/9.8	P/19.8	5	6 m
Goosen 2011	120	120	30	30	60±6.3	62±6.3	15/15	13/17	92% OA	98% OA	26.6±2.6	26.8±2.7	PL/7.8	PL/18	6	1 y
			30	30	60±7.4	62±6.9	15/15	16/14			26.7±3.1	26.1±2.8	AL/7.8	AL/18		
Hart 2005	120	120	60	60	72.4		40/80		100% OA	100% OA	27.6	<35	PL/9-10	PL/20	5	39 m
Kim 2006	70	140	70	70	52	61	53/17		14% OA, 80% ON		25.6		PL/8.8	PL/23.0	5	26m
Martin 2011	79	83	42	41	66.7±10.1	63.1±10.2	12/30	14/27	88% OA	90% OA	30.6±6.1	29.4±5.5	AL/9.5	^*^L/14.8	5	1 y
Mazoochian 2009	51	52	26	26	NA	NA	11/14	9/17	67% OA		26.6±4.5	26.4±3.7	L/8.9	L/14.0	4	3 m
Ogonda 2005	219	219	109	110	67.4±9.8	65.8±10.3	49/60	58/52	98% OA	97% OA	28.2±4.3	28.9±4.3	P/9.50	P/15.81	6	1.5 m
Pospischill 2010	40	40	20	20	61.9	60.6	8/12	12/8	100% OA	100% OA	25.7	25.7	AL/8-10	AL/12	6	3 m
Roy 2010	56	56	25	31	79.5±8.2	84.0±8.1	7/18	4/27	100% FNF		NA	NA	P/<8	P/≥16	4	2 y
Shitama2009	39	39	19	20	58.3±3.0	61.3±10.7	NA	NA	87% DD		23.2±3.6	23.0±3.7	PL/9.0	PL/14.7	5	6 m
Speranza 2007	100	100	46	54	65.0	66.2	20/26	23/31	92% OA	90% OA	28	29	L/7.1	L/12.8	5	6 m
Varela 2013	50	50	25	25	64.8±10.5	63.8±9.7	12/13	13/12	84% OA	88% OA	28.3±3.7	27.8±3.2	L/≤10	L/NA	4	5 y
Yang 2010	110	110	55	55	59.5±13.2	55.8±13.9	26/29	30/25	27% OA	13% OA	23.1±3.2	22.4±3.9	AL/7.49	^*^PL/15.19	4	3 y

Abbreviations: Pts, Patients; BMI, body mass index; FU, follow-up; MI, mini-incisions; SI, standard incisions; OA, osteoarthritis; ON, osteonecrosis; FNF, fenoral neck fracture; DD, Developmental dysplasia; PL, posterolateral; P, posterior; AL, anterolateral; L, lateral; NA, not applicable. * Subgrouping criteria were fundamentally based on the MI approaches except that one trail used two inconsistent but similar approaches between SI and MI subgroups.

The presence of publication bias was assessed by creating a funnel plot which demonstrates the relationship between the sample size of the studies and the precision in estimating the treatment effect. This plot for studies bias can be seen if the plot is widely skewed. A P value <.05 was judged as statistically significant, except where otherwise specified. All statistical analyses were performed using RevMan 5.1 software (Cochrane Collaboration, Oxford, UK).

## Results

### Study Identification and Selection

**Figure 1:**
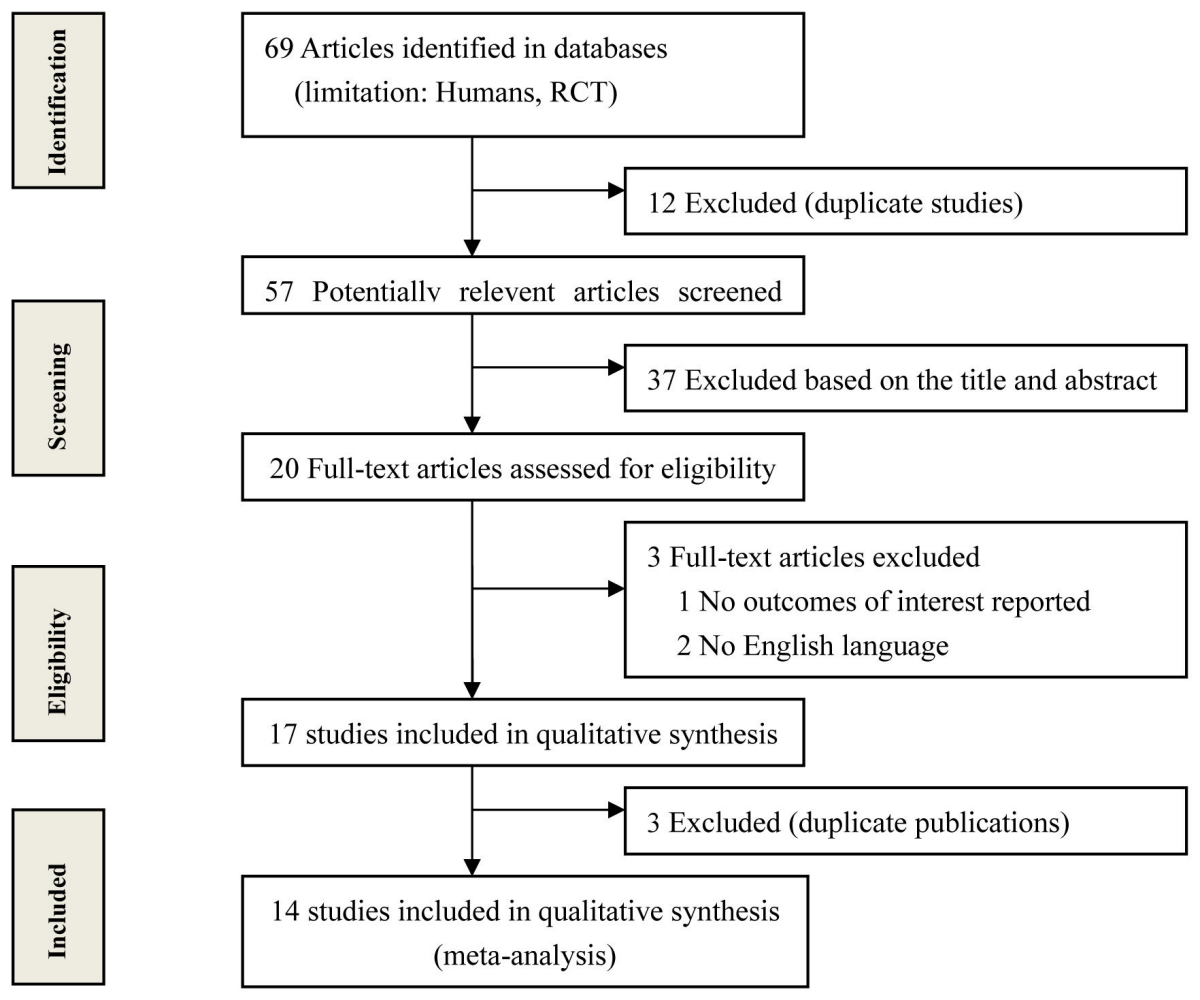
Flow chart of eligibility selection.

An initial database search identified a total of 69 RCTs. 12 RCTs were excluded because of duplicate reportage, and 37 RCTs were excluded based on the titles and abstracts. The remaining 20 full-text articles were reviewed for more detailed evaluation; 3 of them were further excluded because one study did not report outcomes of interest [20], one study was in Chinese [21], and the last one was in German [22]. Three additional RCTs were excluded because of repeated data used [23–25]. Finally, 14 RCTs that met our inclusion criteria were included in the present meta-analysis [26–39]. The flowchart of inclusion process is shown in Figure 1.

### Characteristics of the RCTs

**Figure 2:**
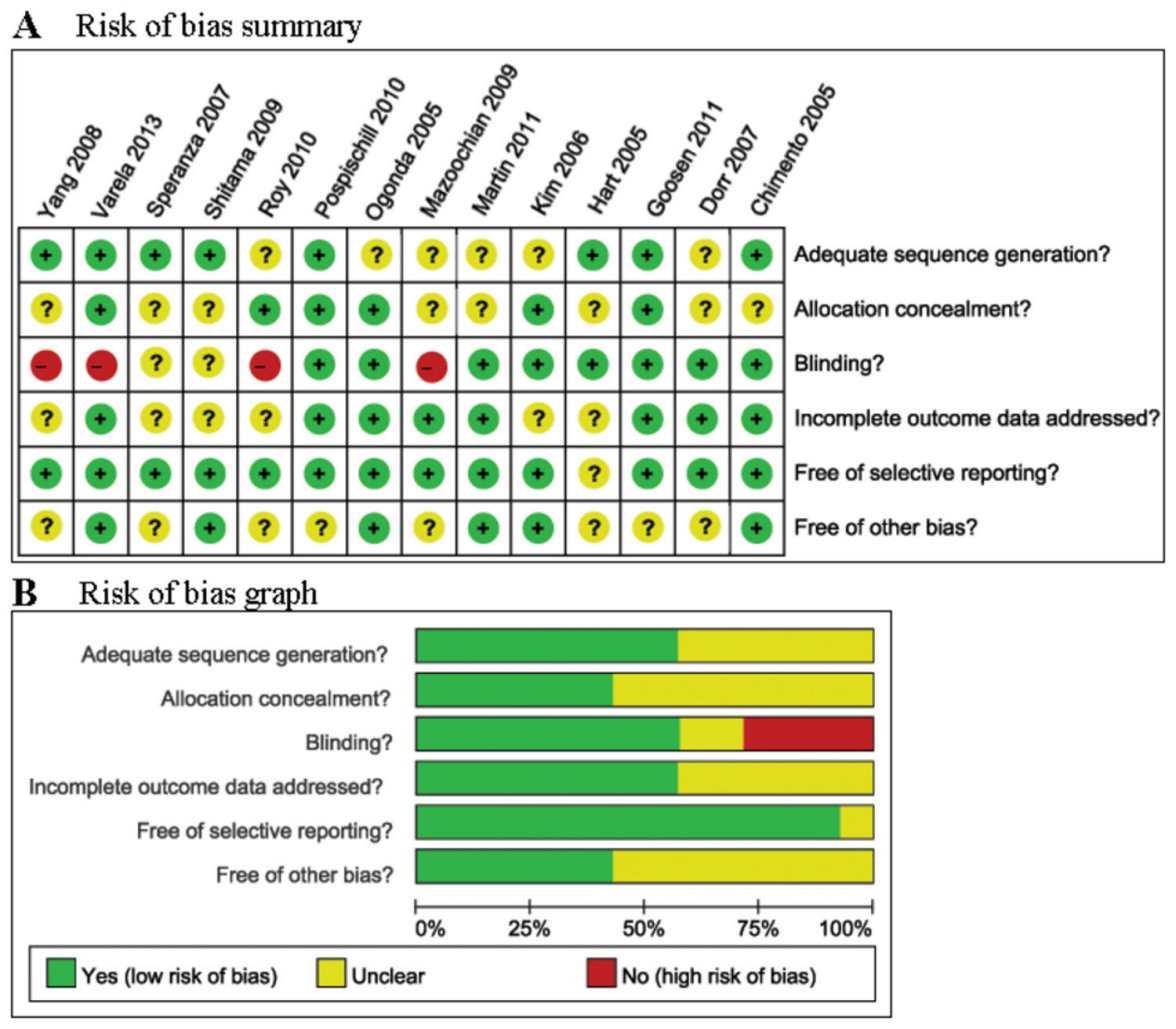
Risk of bias summary and graph.

The main characteristics of the 14 RCTs included in the present meta-analysis are summarized in Table 1. These studies were published between 2005 and 2013. The sample size of the RCT ranged from 39 to 120 (total 1,174). Consistent baselines were observed for all patients. Among the 14 studies included here, the main preoperative diagnoses were osteoarthritis, osteonecrosis, femoral neck fracture and developmental dysplasia. Outcomes of interest were reported in all the studies. The quality of the included studies was assessed by the Cochrane Collaboration’s tool for risk of bias. As is shown in Figure 2, all the trials were found to be of medium risk of bias. The median Jadad score of the studies included was 5 (range of 4 to 6) (Table 1).

Of the 14 studies, 3 included surgical interventions via the posterior approach [27,29,35], 5 involved the posterolateral approach [26,28,30,31,36], 3 the lateral approach [33,37,38] and 4 the anterolateral approach [30,32,34,39]. Among them, the study [30] by Goosen et al. involved both the anterolateral and the posterior approaches. Follow-ups of clinical outcomes and complications ranged between 6 weeks and 80 months. 

### Surgical Outcomes: surgical duration, blood loss, pain and length of hospital stay

**Figure 3:**
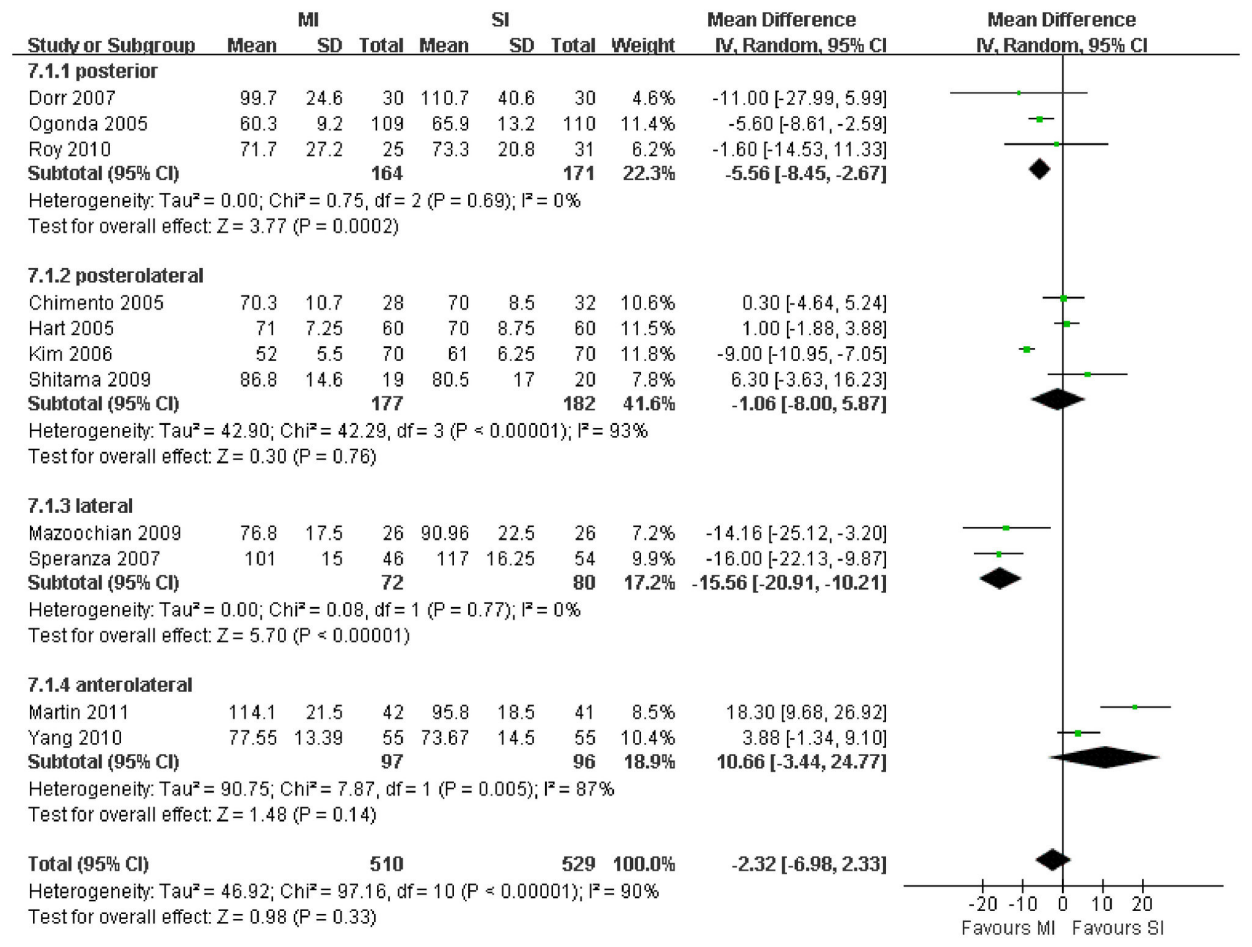
Meta-analysis of randomized controlled trials evaluating surgical duration.

11 studies with a total of 1,039 patients were suitable for meta-analysis of surgical duration. Generally there was no significant difference in this respect between MI and SI THA (WMD, -2.32 min; 95% CI, -6.98 to 2.33; p=.33; Figure 3). The test for heterogeneity was significant (I^2^ =90%). Subsequently, we performed sensitivity analyses to explore potential source of heterogeneity. Exclusion of 2 studies [32,39] that had different interventions (AL vs L, AL vs PL) in the anterolateral subgroup or 2 studies [33,37] whose data were collected from a skewed distribution sample in the lateral subgroup yielded similar results (WMD, -2.72 min; 95% CI, -7.28 to 1.83; P =.24) also with a significant heterogeneity (I^2^ = 86%). However, there was a significant difference between the posterior subgroup (WMD, -5.56 min; 95% CI, -8.45 to -2.67; P<.001) and the lateral subgroup (WMD, -15.56 min; 95% CI, -20.91 to -10.21; P<.001) with no evidence of heterogeneity among the studies. 

**Figure 4:**
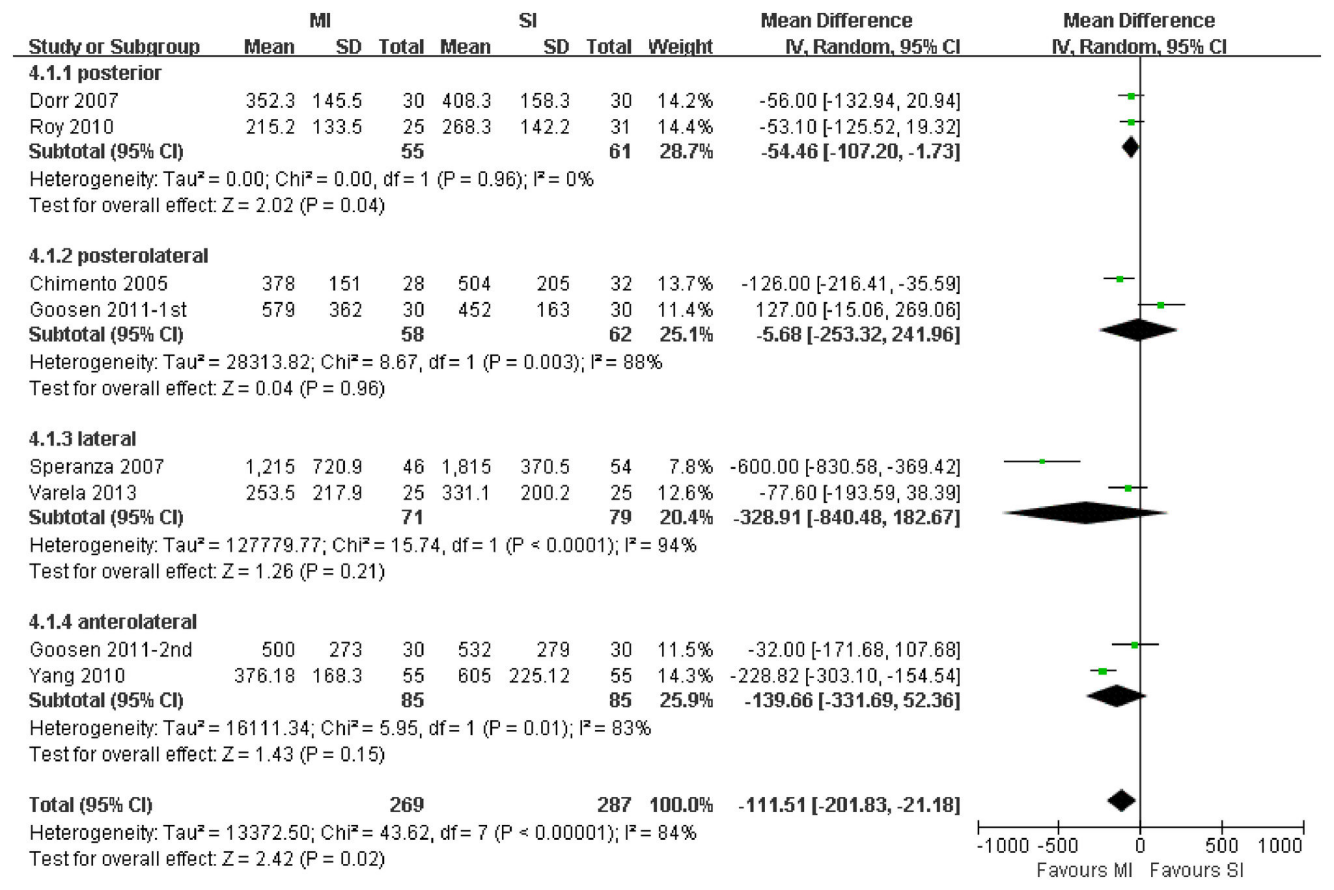
Meta-analysis of randomized controlled trials evaluating blood loss.

11 trials with a total of 556 patients were suitable for meta-analysis of total blood loss. MI reduced the total blood loss compared with SI THA (WMD, -111.51 ml; 95% CI, -201.83 to -21.18; p=.02; Figure 4). The test for heterogeneity was significant (I^2^ = 84%). Sensitivity analyses were performed after 1 study [37] with low quality had been excluded, yielding similar results (WMD, -73.59 ml; 95% CI, -147.61 to 0.44; p=.05). Meta-analysis of the posterior MI and SI subgroups had a significant difference in total blood loss (WMD, -54.46 ml; 95% CI, -107.20 to -1.73; P=.04) with no evidence of heterogeneity (I^2^ = 0%). 

**Figure 5:**
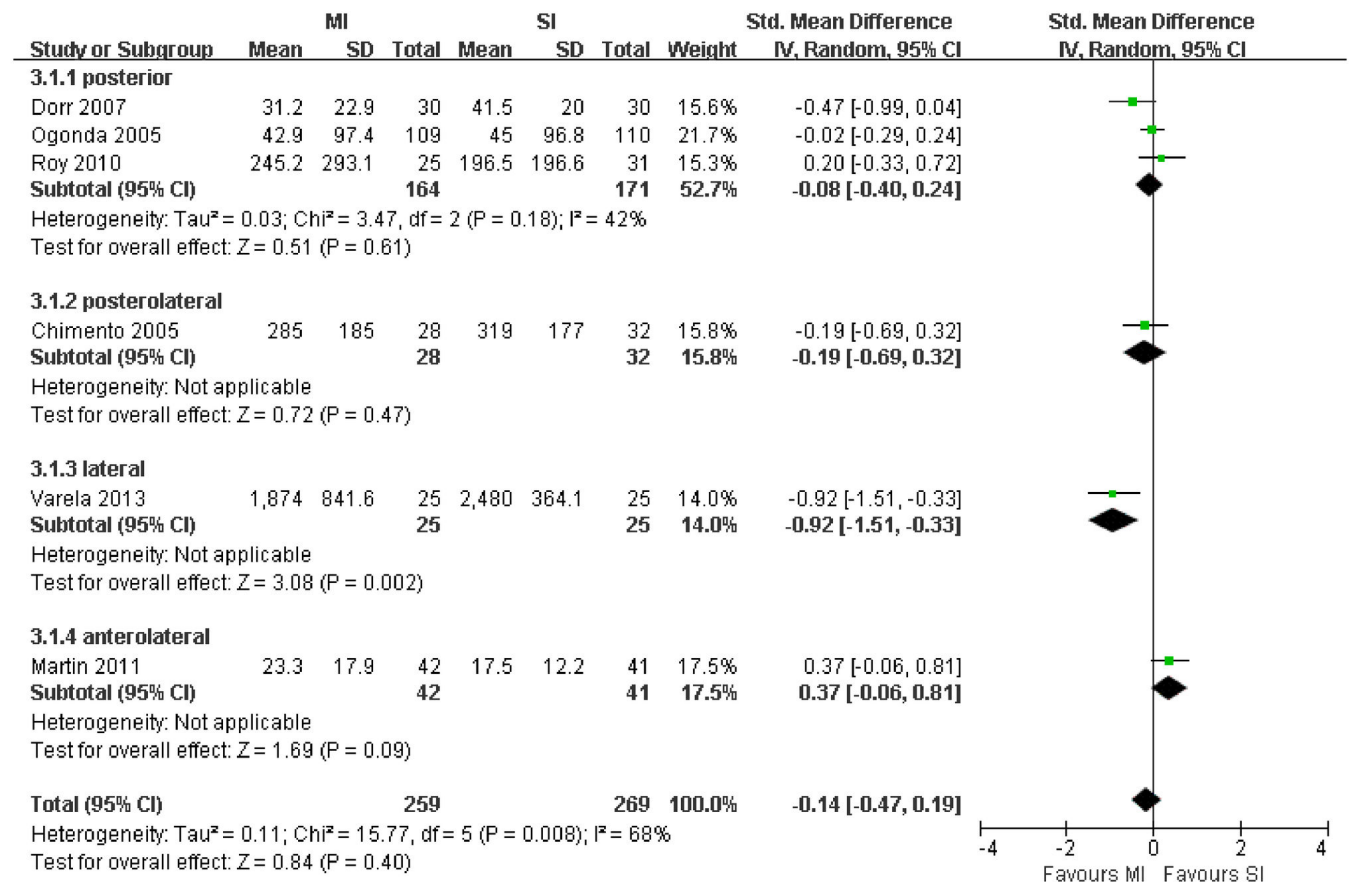
Meta-analysis of randomized controlled trials evaluating doses of pain medication.

6 RCTs with a total of 528 patients were suitable for meta-analysis of doses of pain medication. There was no significant difference with regard to doses of pain medication between patients receiving MI THA and those receiving SI THA (SMD, -0.14; 95% CI, -0.47 to 0.19; p=.40; Figure 5). The heterogeneity was moderate (I^2^ = 68%). Sensitivity analyses yielded similar results after exclusion of 2 studies [32,38] that did not use morphine consumption as doses of pain medication (SMD, -0.09; 95% CI, -0.32 to 0.14; p=.43). Similarly, there was not a significant difference between the posterior MI and SI subgroups in morphine consumption (WMD, -0.08; 95% CI, -0.40 to 0.24; P=.61) with low heterogeneity (I^2^ = 42%). 

**Figure 6:**
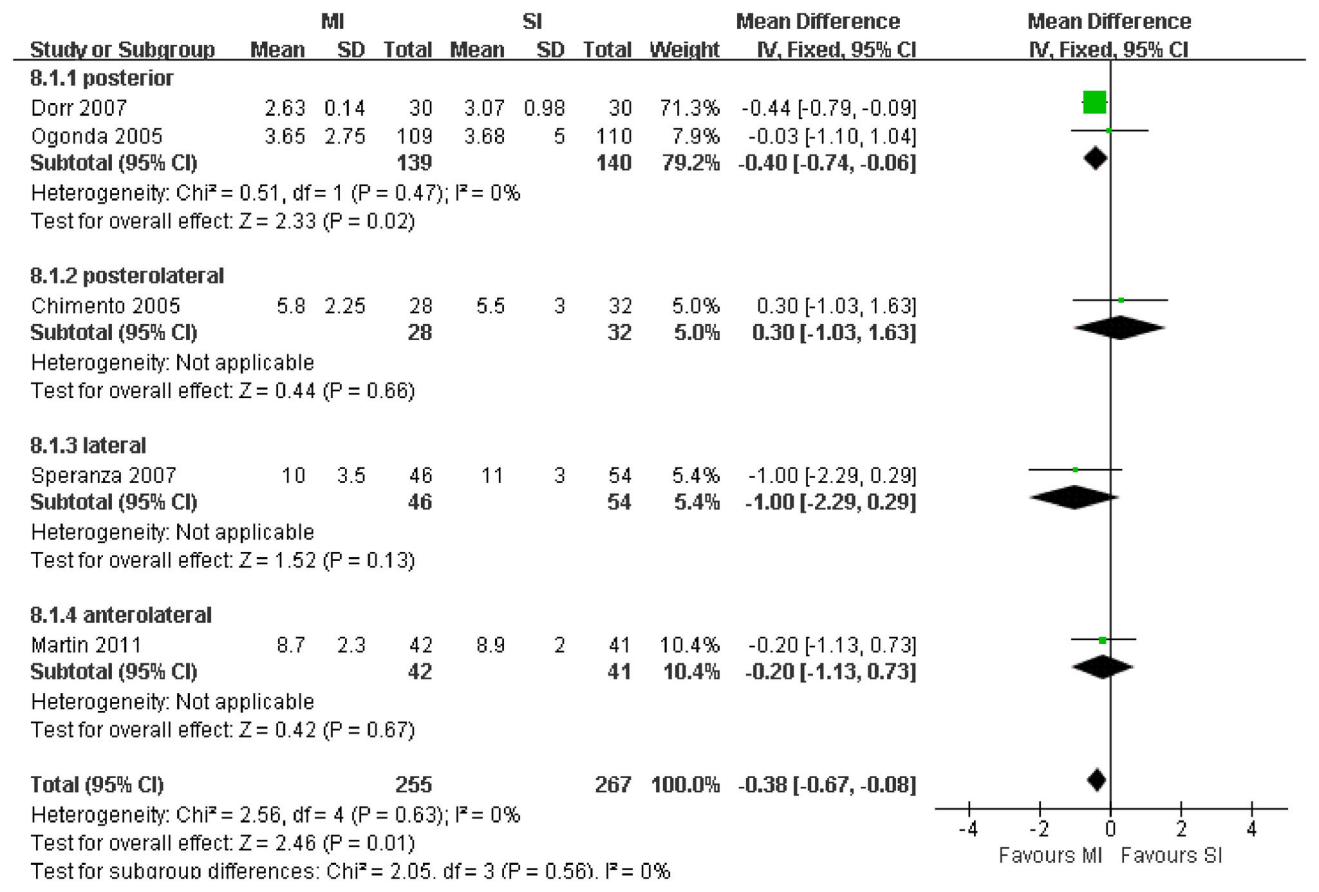
Meta-analysis of randomized controlled trials evaluating length of hospital stay.

The aggregated results of 5 studies with a total of 522 patients suggest that MI significantly reduced the length of hospital stay for patients receiving THA (WMD, -0.38 days; 95% CI, -0.67 to -0.08; p=.01; Figure 6). The subgroup analyses revealed that there was a significant difference in the length of hospital stay between the posterior subgroup and the control subgroup (2 RCTs; WMD, -0.40 days; 95% CI, -0.74 to -0.06; p=.002) but no significant differences between the other 3 subgroups and their control subgroups (Figure 6). 

### Functional Outcome: HHS

**Figure 7:**
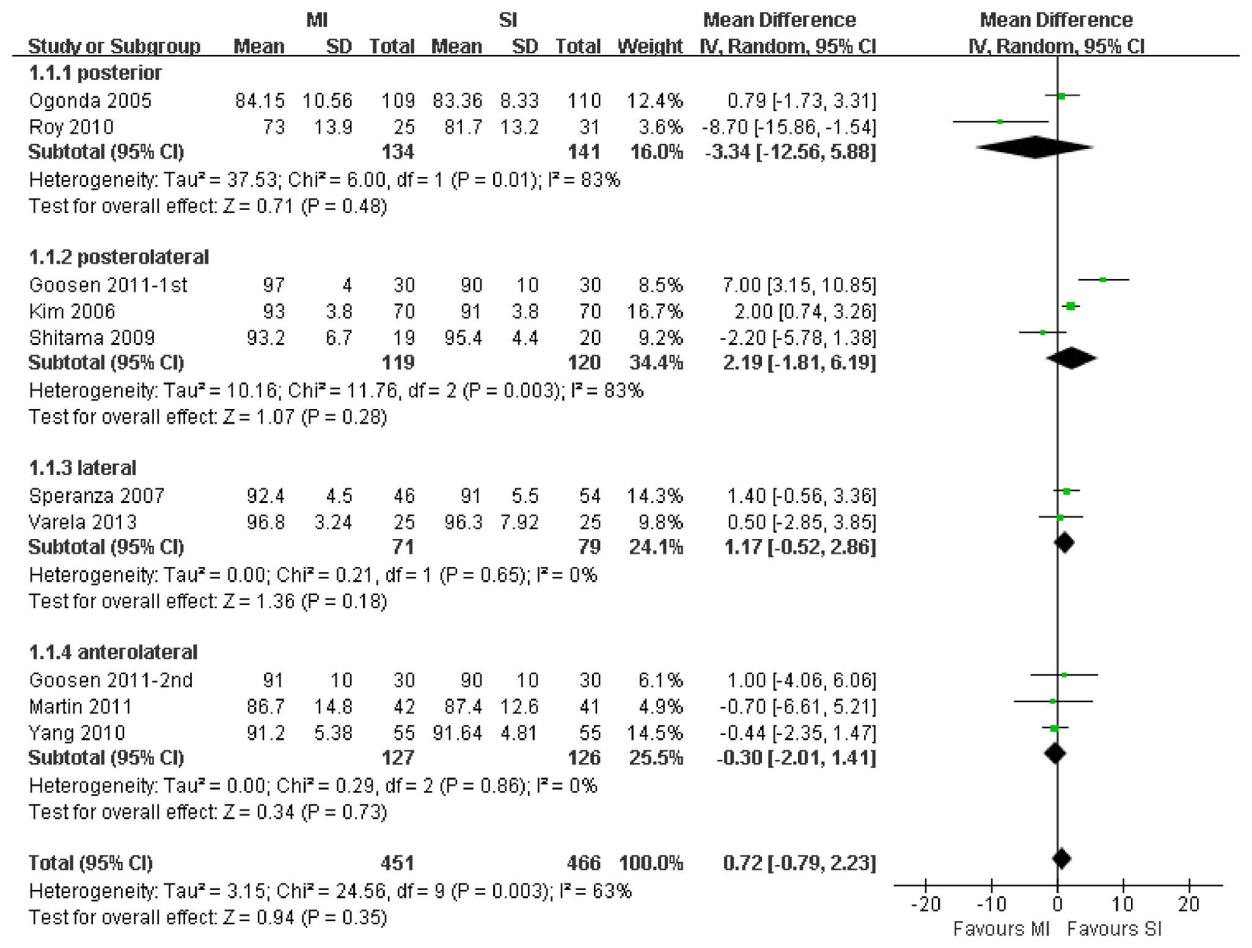
Meta-analysis of randomized controlled trials evaluating HHS.

10 trails with a total of 917 patients who were available at the last follow-up for HHS data were suitable for meta-analysis of HHS. Figure 7 shows the results of HHS at the last follow-up. There was no significant difference between MI and SI THA groups (WMD, 0.72; 95% CI, -0.79 to 2.23; P=.35). The results did not change after sensitivity analysis. The forest plot showed the HHS for MI THA was not significantly higher than that for SI THA in each subgroup (P>.05).

### Radiological Outcomes

There were no significant differences between MI and SI THA groups in the outliers of acetabular cup abduction (5 trails; OR, 0.86; 95% CI, 0.52 to 1.42; p=.56), outliers of acetabular cup anteversion (2 trails; OR, 1.19; 95% CI, 0.51 to 2.79; p=.69), outliers of femoral prosthesis position (9 trails; OR, 0.75; 95% CI, 0.45 to 1.24; p=.27), femoral offset (3 trails; WMD, 0.36; 95% CI, -0.12 to 0.76; p=.15), leg-length discrepancy (4 trails; WMD, -0.24; 95% CI, -1.76 to 1.28; p=.75). Notably, there were a significant difference between the posterolateral MI and SI subgroups THA groups regarding the femoral offset (1 trails; WMD, 3.00 mm; 95% CI, 0.40 to 5.60; p=.02), but there were no such significant differences between other MI and SI subgroups (Table 2).

**Table 2:** Meta-analysis of randomized controlled trials evaluating radiological outcomes.

Radiological Outcomes	Trails, No.	Patients, No.	OR/WMD (Fixed, 95%CI)	P-value
1 outliers of acetabular cup abduction	5	588	0.86[0.52, 1.42]	0.56
1.1 posterior subgroup	1	219	0.82[0.40, 1.70]	0.60
1.2 posterolateral subgroup	1	140	1.22[0.35, 4.19]	0.75
1.3 lateral subgroup	2	150	0.92[0.34, 2.48]	0.86
1.4 anterolateral subgroup	1	79	0.55[0.12, 2.48]	0.44
2 outliers of acetabular cup anteversion	2	223	1.19[0.51, 2.79]	0.69
2.1 posterior subgroup	0	0	Not estimable	Not estimable
2.2 posterolateral subgroup	1	140	1.19[0.38, 3.72]	0.77
2.3 lateral subgroup	0	0	Not estimable	Not estimable
2.4 anterolateral subgroup	1	83	1.20[0.34, 4.29]	0.78
3 outliers of femoral prothesis position	9	934	0.75[0.45, 1.24]	0.27
3.1 posterior subgroup	1	219	0.36[0.09, 1.40]	0.14
3.2 posterolateral subgroup	3	320	0.92[0.39,2.16]	0.85
3.3 lateral subgroup	3	202	0.55[0.20, 1.48]	0.24
3.4 anterolateral subgroup	2	193	1.34[0.45, 4.02]	0.60
4 femoral offset	3	283	0.36[-0.12, 0.76]	0.16
4.1 posterior subgroup	1	60	2.20[-0.85, 5.25]	0.16
4.2 posterolateral subgroup	1	140	3.00[0.40, 5.60]	0.02
4.3 lateral subgroup	0	0	Not estimable	Not estimable
4.4 anterolateral subgroup	1	83	0.20[-0.25, 0.65]	0.39
5 leg-length discrepancy	4	320	-0.24[-1.76, 1.28]	0.75
5.1 posterior subgroup	1	60	-1.60[-4.87, 1.67]	0.34
5.2 posterolateral subgroup	2	200	-0.01[-1.91, 1.88]	0.99
5.3 lateral subgroup	0	0	Not estimable	Not estimable
5.4 anterolateral subgroup	1	60	0.80[-3.28, 4.88]	0.70

Abbreviations: NO, number; OR, odds radio; WMD, weighted mean difference.

### Complications

There were no significant differences between MI and SI THA groups in infection (12 trails; RD, 0.01; 95% CI, -0.01 to 0.03; p=.25), dislocation (11 trails; RD, 0.00; 95% CI, -0.01 to 0.02; p=.60), nerve injury (8 trails; RD,0.01; 95% CI, -0.01 to 0.03; p=.24), proximal femoral fracture (8 trails; RD, 0.01; 95% CI, -0.02 to 0.04; p=.61), DVT (4 trails; RD, -0.01; 95% CI, -0.04 to 0.01; p=.25), component loosening (5 trails; RD, 0.01; 95% CI, -0.02 to 0.04; p=.38), revision (5 trails; RD, 0.02; 95% CI, -0.03 to 0.07; p=.40), or heterotopic ossification (5 trails; RD, -0.02; 95% CI, -0.07 to 0.04; p=.58). Subgroup meta-analyses showed no such significant differences either between all the subgroups (p>.05) (Table 3).

**Table 3:** Meta-analysis of randomized controlled trials evaluating complications outcomes.

Complications Outcomes	Trails, No.	Patients, No.	RD (Fixed, 95%CI)	P-value
1 infection	12	1039	0.01[-0.01, 0.03]	0.25
1.1 posterior subgroup	3	335	0.02[-0.01, 0.05]	0.21
1.2 posterolateral subgroup	4	359	0.01[-0.02, 0.03]	0.65
1.3 lateral subgroup	3	202	0.01[-0.03, 0.05]	0.61
1.4 anterolateral subgroup	2	143	0.00[-0.05, 0.05]	1.00
2 dislocations	11	959	0.00[-0.01, 0.02]	0.60
2.1 posterior subgroup	3	335	0.00[-0.02, 0.02]	1.00
2.2 posterolateral subgroup	3	239	0.02[-0.02, 0.06]	0.38
2.3 lateral subgroup	2	152	0.00[-0.04, 0.04]	1.00
2.4 anterolateral subgroup	3	233	0.00[-0.03, 0.03]	1.00
3 nerve injury	8	680	0.01[-0.01, 0.03]	0.24
3.1 posterior subgroup	1	60	0.00[-0.06, 0.06]	1.00
3.2 posterolateral subgroup	4	380	0.01[-0.02, 0.04]	0.43
3.3 lateral subgroup	1	97	0.04[-0.03, 0.11]	0.22
3.4 anterolateral subgroup	2	143	0.00[-0.04, 0.04]	1.00
4 proximal femoral fracture	8	529	0.01[-0.02, 0.04]	0.61
4.1 posterior subgroup	1	60	-0.03[-0.14, 0.08]	0.55
4.2 posterolateral subgroup	3	219	0.01[-0.03, 0.04]	0.60
4.3 lateral subgroup	2	150	0.00[-0.05, 0.05]	0.97
4.4 anterolateral subgroup	2	100	0.04[-0.06, 0.14]	0.43
5 DVT	4	462	-0.01[-0.04, 0.01]	0.25
5.1 posterior subgroup	2	279	-0.01[-0.04, 0.01]	0.31
5.2 posterolateral subgroup	0	0	Not estimable	Not estimable
5.3 lateral subgroup	1	100	0.00[-0.04, 0.04]	1.00
5.4 anterolateral subgroup	1	83	-0.02[-0.09, 0.04]	0.46
6 component loosening	5	453	0.01[-0.02, 0.04]	0.38
6.1 posterior subgroup	0	0	Not estimable	Not estimable
6.2 posterolateral subgroup	2	200	-0.01[-0.04, 0.02]	0.56
6.3 lateral subgroup	0	0	Not estimable	Not estimable
6.4 anterolateral subgroup	3	253	0.03[-0.01, 0.08]	0.17
7 revision	5	290	0.02[-0.03, 0.07]	0.40
7.1 posterior subgroup	1	60	0.00[-0.13, 0.13]	1.00
7.2 posterolateral subgroup	2	120	0.00[-0.06, 0.07]	0.97
7.3 lateral subgroup	1	50	0.03[-0.07, 0.07]	1.00
7.4 anterolateral subgroup	1	60	0.10[-0.04, 0.24]	0.15
8 heterotopic ossification	5	373	-0.02[-0.07, 0.04]	0.58
8.1 posterior subgroup	1	60	0.00[-0.09, 0.09]	1.00
8.2 posterolateral subgroup	1	60	0.00[-0.17, 0.17]	1.00
8.3 lateral subgroup	0	0	Not estimable	Not estimable
8.4 anterolateral subgroup	3	253	-0.02[-0.10, 0.05]	0.51

Abbreviations: NO, number; RD, risk difference.

### Publication Bias

**Figure 8:**
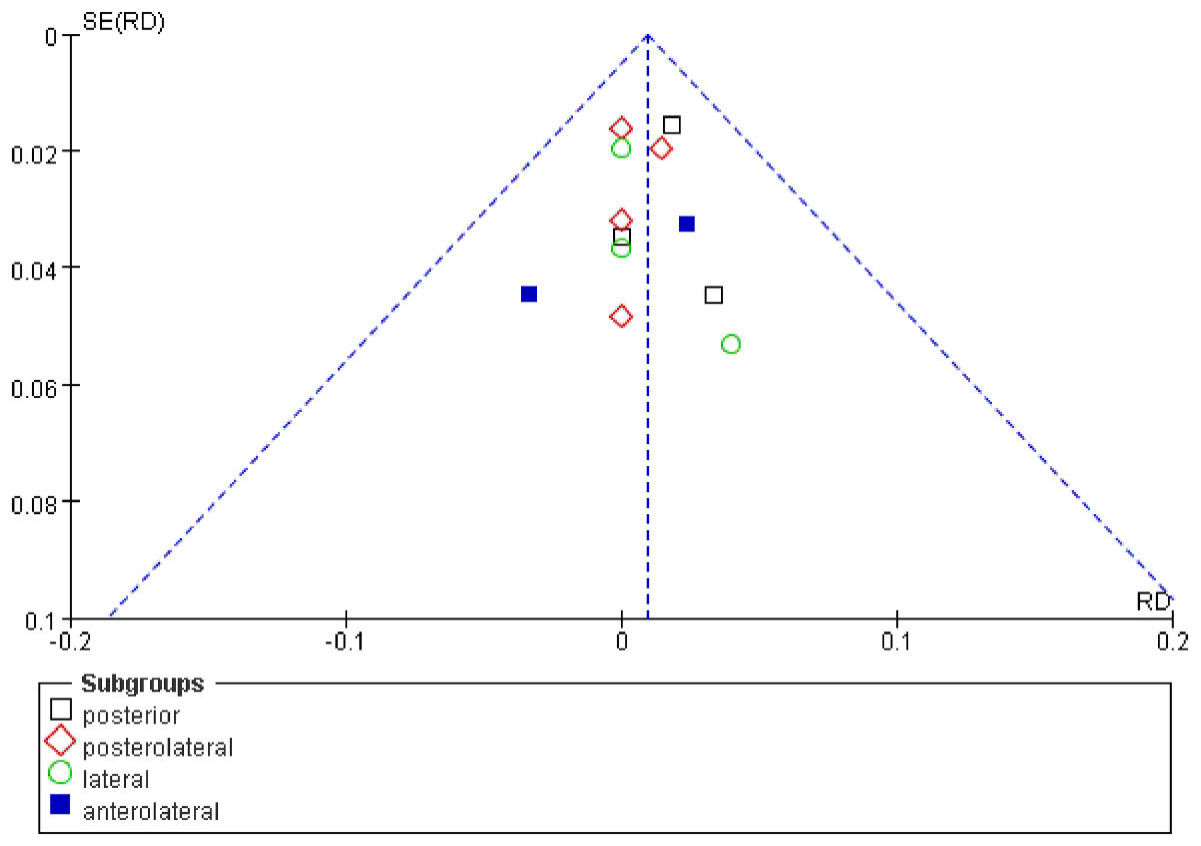
Funnel plot showing minimal publication bias of the complication outcome- infection. SE(RD), standard error (risk differences).

Because of sufficient numbers of included studies (>10), funnel plot was used to assess publication bias. Infection in complication outcomes was pooled for the funnel plot analysis, indicating minimal evidence of publication bias (Figure 8). 

## Discussion

The pooled results with significant heterogeneity from the present meta-analysis of 14 RCTs using a random-effects model suggest that MI THA may reduce just blood loss compared with SI THA in general. Specifically, posterior MI was revealed to have reduced significantly surgical duration, blood loss, and length of hospital stay but have had no significant impact on pain or HSS compared with posterior SI in the subgroup meta-analysis, indicating that posterior MI is advantageous over SI as an alternative approach for patients undergoing THA. No obvious differences were observed between MI and SI in overall meta-analysis or in subgroup analyses regarding radiological and complication outcomes. 

No significant differences were revealed by the present overall meta-analysis in surgical duration between MI and SI groups. However, strong statistical heterogeneity was observed among the 14 RCTs. It is not surprising given the differences in surgical approaches adopted in both MI and SI THA. Since it was difficult to draw a reliable conclusion based on results of high heterogeneity about a possible difference in surgical duration between the two procedures, we conducted subgroup analyses which demonstrated with no heterogeneity that posterior MI was associated with a reduction (5.56 min) in surgical duration. Compared with other MI approaches, posterior MI may be the simplest and mostly used, resulting in shortened operating time. As Sculco et al. [23] suggested, the posterior approach may be the most appropriate approach to adopt since it is familiar to most surgeons. However, Cheng et al. [8] doubted the clinical value of an average reduction of 4.73 min in surgical duration which had been achieved in their study. To the best of our knowledge, reduction in the operative time, no matter it averaged only 5.56 min according to our results, can decrease the risk of anesthesia in operation, especially for senior patients undergoing THA who had suffered from some basic diseases. It must be pointed out that the reduction in operative time (15.56 min) in lateral MI subgroup might be a doubtful result which needs to be tested in the future study, because the estimates were based on the data which had skewed distribution in RCTs [33,37]. 

In line with previous studies [7,8], a major finding from our overall meta-analysis was a significantly lower total blood loss in MI THA than in SI THA. The advantage of less blood loss may result from more limited exposure, less tissue injury and reduced surgery time in MI surgical procedures. Although sensitivity analyses yielded similar results, this finding should be interpreted cautiously because of significant heterogeneity across study designs. Another interesting finding was that merely posterior MI, not other mini-incisions, significantly reduced the blood loss. This is probably because posterior MI was completed in muscular spaces very different from those in other locations. Thus, it is worthwhile to study this specific MI approach for THA — posterior mini-incision — regarding the blood loss. Moreover, because of less visualization of surgical field when a small incision used, it is not clear if hidden blood loss may increase or may have any impact on the recovery time.

Our overall and subgroup meta-analyses indicated that MI led to no statistically significant benefit in doses of pain medication (measured in milligrams of morphine or metamizol). Using the VAS of pain in a retrospective study, Wong et al. [40] and Dorr et al. [29] found less pain in MI THA group. Moskal et al. [7] and Smith et al. [9] also came to the same conclusion in their meta-analysis. Although reduced pain was one of the benefits that had been cited for MI approach, it is problematic and not sound to estimate pain by subjective assessment of VAS. Based on analgesic consumption, our results indicated MI THA did not result in better early pain control, which was in agreement with Pour et al. [41], de Beer et al. [42] and Asayama et al. [43]. Remarkably, one RCT [38] included in our meta-analysis of the lateral subgroup showed a significant reduction in metamizol intaking. We believe, however, this finding was not powerful because of the different analgesic regime they used and a small number of subjects in their study which likely led to type II statistical error [44].

Consistent with previous overall meta-analyses [8,9], we found the length of hospital stay was significantly shorter among MI THA patients. Similarly, an interesting finding was that only posterior MI, rather than other mini-incisions, resulted in significantly shorter hospital stay in our subgroup meta-analysis with no statistically heterogeneity. A reduction in length of hospital stay provides more tangible benefits for both patients and surgeons than a reduction in surgical duration and blood loss. This is of great importance since shorter length of hospital stay may reduce the risk of iatrogenic infection and result in a substantial financial benefit. Since none of the identified RCTs provided a cost-effectiveness analysis, this may be an interesting focus for future studies [45].

As for hip functional score, our results demonstrated that MI THA resulted in similar HHS compared with SI THA, consistent with some studies [6,8,9] but contrary to other reports [7,11]. But the findings about HHS by the previous meta-analyses were not strong enough because they included many lower quality non-randomised studies. Furthermore, the HHS system was not adopted by all the trails and the data were collected at different stages of follow-up in many clinical studies. Consequently, the corresponding synthesized results in meta-analyses comparing MI and SI THA had a significant heterogeneity, including ours. Thus, the conclusion about HHS must be non-conclusive in nature. Disappointingly, no consistent and sufficient indexes about hip functional recovery can be collected for a meta-analysis comparing the current two THA procedures because no uniform hip functional evaluation system has been agreed upon by all the clinical researchers. Other relevant evaluation indexes like Western Ontario and McMaster Universities Arthritis Index (WOMAC) and Oxford hip score are also used in some clinical trails. Ogonda et al. [27] used WOMAC scores and Oxford hip score but found no difference between the two procedures. 

Those who doubted MI THA suggested that poor exposition and visualization of important landmarks could precipitate inadequate orientation of the components or increase complications. However, our meta-analysis of radiological outcomes and complications indicated that there were no significant differences between the two THA procedures. Our subgroup analysis found that the average femoral offset in one study [26] was significantly increased by 3 mm in the posterolateral MI group, though it was reported that decreased femoral offset improved soft tissue tension and decreased impingement, resulting in better hip stability [46]. In fact, the result above can not be conclusive because it was derived from only 140 participants in a trial that was not adequately powered to examine the effect. Our meta-analysis found no significant difference in nerve injury between MI and SI THA, in agreement with one previous study [13] but in disagreement with a systematic review and meta-analysis [9] that showed an association of MI THA with a significantly increased risk of transient lateral femoral cutaneous nerve palsy. 

The overall pooled estimates from our meta-analysis of 14 RCTs showed significant statistical heterogeneity, though the trials we had included had clinical homogeneity. The significant statistical heterogeneity (I^2^ >50%) among the trials is not only the major limitation of the present study, but also a common limitation in systematic reviews and meta-analyses. But we think it is clearly of great interest and value to determine the causes of heterogeneity among the results of the studies. We found that one major heterogeneity among the results in the present study might have resulted from the four different surgical approaches (posterior / posterolateral / lateral / anterolateral approach) used in the mini-incision total hip arthroplasty. It was just this sort of heterogeneity that triggered us to perform further sub-group analyses and had the most interesting finding of the present study. The posterior mini-incision THA had perioperative advantages of reduced surgical duration, less blood loss and shorter hospital stay with no heterogeneity (I^2^=0, respectively) and without increasing the risk of component malposition or complications, which is a new reliable evidence in the present meta-analysis we conducted. Other factors which might have led to the heterogeneity among the results included population characteristics, preoperative diagnosis, treatment protocol, prosthesis type, expertise or skills of surgeons or hospitals, study design varied considerably across the studies included, and so on. In brief, we did not perform further sub-group analyses of these factors either because of limited clinical significance or because of slim feasibility. To make up for the inevitable heterogeneity which might have a bias effect on the results of our analyses, we made sensitivity analyses to testify the robustness of our main findings from overall meta-analysis. The sensitivity analyses showed that our main findings were still stable from our overall meta-analysis, though they should be interpreted with caution due to the significant heterogeneity among studies. 

In addition, this meta-analysis has other several limitations that should be taken into account. First, most of the 14 RCTs in our analysis had a modest sample size. Overestimation of the treatment effect is more likely in smaller trials compared with larger samples. Secondly, our study failed to discuss two important outcomes to evaluate the two surgical procedures we were interested in, radiation exposure and cost. This is because, unfortunately, the RCTs currently available comparing the two methods of THA did not report enough data regarding the two outcomes. The high accuracy in hip reconstruction should be based on the optimizing location of implants which usually depends on conventional X-ray radiographs or computed tomography [47]. However, own to limited visibility for anatomical landmarks and vital structures in MI THA, this minimally invasive procedure may potentially induce a higher radiation exposure compared with SI THA and may consequently increase costs for component positioning. This is an interesting issue but was mostly ignored in relevant researches. Thus, further research should pay more attention on the two significant outcomes. Finally, we were unable to assess the impact of MI THA on other clinically meaningful end points, such as time of recovery, gait and service life of prosthesis, because of sparse and inconsistent data reported across trials. 

It is an urgent need to standardize clinical THA protocols (involving consistent definitions of population characteristics, surgical approaches, prosthesis types, evaluation measures, rehabilitation program, etc.) since great variability exists in the current literature. Next, effects of other coexisting confounders need to be excluded in synergistic meta-analysis of a factor of interest to eliminate heterogeneity as much as possible. Finally, although posterior MI THA is generally considered safe and well tolerated, its cost-effectiveness should be a concern for future studies.

## Conclusions

In conclusion, current evidence suggests that posterior MI THA has definite advantages over SI THA. It may result in significantly shorter surgical duration, less blood loss and shorter hospital stay, but not increase risk of component malposition or complications. However, since the sample sizes of all the relevant RCTs were not sufficient enough, this encouraging finding should not be a finalized conclusion. Further large-scale, well-designed RCTs on this topic are still needed. In such future studies, long-term outcomes like recovery time should also be taken into consideration.

## Supporting Information

Checklist S1 **PRISMA Checklist.**
(DOC)

Table S1 **Cochrane Collaboration’s tool for assessing risk of bias.**
(DOC)

Table S2 **Modified Jadad Scale with eight items.**
(DOC)
