# Supplementary material for: Mini-Incision versus Standard Incision Total Hip Arthroplasty Regarding Surgical Outcomes: A Systematic Review and Meta-Analysis of Randomized Controlled Trials
Source: PLoS One. 2013 Nov 12;8(11):e80021. doi: 10.1371/journal.pone.0080021 (PMC3827164; doi:10.1371/journal.pone.0080021)
Supplement: Table S1 — Cochrane Collaboration’s tool for assessing risk of bias. (DOC) [file pone.0080021.s002.doc]

| **Bias domain** | **Support for judgment** | **Review authors’ judgment (assess as low, unclear or high risk of bias)** |
| --- | --- | --- |
| **Sequence generation** | Describe the method used to generate the allocation sequence in sufficient detail to allow an assessment of whether it should produce comparable groups. | Was the allocation sequence adequately generated? |
| **Allocation concealment** | Describe the method used to conceal the allocation sequence in sufficient detail to determine whether intervention allocations could have been foreseen in advance of, or during, enrolment | Was allocation adequately concealed? |
| **Blinding** | Describe all measures used, if any, to blind study participants and personnel from knowledge of which intervention a participant received. Provide any information relating to whether the intended blinding was effective. | Was knowledge of the allocated intervention adequately prevented during the study? |
| **Incomplete outcome data** | Describe the completeness of outcome data for each main outcome, including attrition and exclusions from the analysis. State whether attrition and exclusions were reported, the numbers in each intervention group (compared with total randomized participants), reasons for attrition/ exclusions where reported, and any re-inclusions in analyses performed by the review authors. | Were incomplete outcome data adequately addressed? |
| **Reporting bias** | State how the possibility of selective outcome reporting was examined by the review authors, and what was found. | Are reports of the study free of suggestion of selective outcome reporting? |
| **Other sources**  **of bias** | State any important concerns about bias not covered in the other domains in the tool. If particular questions/items were pre-specified in the review’s protocol, responses should be provided for each question/item. | Was the study apparently free of other problems that could put it at a high risk of bias? |
