# Supplementary material for: Mini-Incision versus Standard Incision Total Hip Arthroplasty Regarding Surgical Outcomes: A Systematic Review and Meta-Analysis of Randomized Controlled Trials
Source: PLoS One. 2013 Nov 12;8(11):e80021. doi: 10.1371/journal.pone.0080021 (PMC3827164; doi:10.1371/journal.pone.0080021)
Supplement: Table S2 — Modified Jadad Scale with eight items. (DOC) [file pone.0080021.s003.doc]

| **Items assessed** | **Response** | **Score** |
| --- | --- | --- |
| Was the study described as randomized? | YES | +1 |
|  | NO | 0 |
| Was the method of randomization appropriate? | YES | +1 |
|  | NO | -1 |
|  | NOT DESCRIBED | 0 |
| Was the study described as blinded?* | YES | +1 |
|  | NO | 0 |
| Was the method of blinding appropriate? | YES | +1 |
|  | NO | -1 |
|  | NOT DESCRIBED | 0 |
| Was there a description of withdrawals and dropouts? | YES | +1 |
|  | NO | 0 |
| Was there a clear description of the inclusion/exclusion criteria? | YES | +1 |
|  | NO | 0 |
| Was the method used to assess adverse effects described? | YES | +1 |
|  | NO | 0 |
| Was the method of statistical analysis described? | YES | +1 |
|  | NO | 0 |

*Double-blind RCT obtains 1 score; single-blind RCT obtains 0.5 score.
